# Anticancer Ribosomally Synthesized and Post-Translationally Modified Peptides from Plants: Structures, Therapeutic Potential, and Future Directions

**DOI:** 10.3390/cimb47010006

**Published:** 2024-12-26

**Authors:** Hyeon-Jeong Hwang, Youngsang Nam, Chanhee Jang, Eun La Kim, Eun Seo Jang, Yeo Jin Lee, Seoung Rak Lee

**Affiliations:** College of Pharmacy, Research Institute for Drug Development, Pusan National University, Busan 46241, Republic of Korea

**Keywords:** phytochemicals, ribosomally synthesized and post-translationally modified peptides, chemical structure, anticancer activity, biosynthesis

## Abstract

Cancer remains a significant medical challenge, necessitating the discovery of novel therapeutic agents. Ribosomally synthesized and post-translationally modified peptides (RiPPs) from plants have emerged as a promising source of anticancer compounds, offering unique structural diversity and potent biological activity. This review identifies and discusses cytotoxic RiPPs across various plant families, focusing on their absolute chemical structures and reported cytotoxic activities against cancer cell lines. Notably, plant-derived RiPPs such as rubipodanin A and mallotumides A–C demonstrated low nanomolar IC_50_ values against multiple cancer cell types, highlighting their therapeutic potential. By integrating traditional ethnobotanical knowledge with modern genomic and bioinformatic approaches, this study underscores the importance of plant RiPPs as a resource for developing innovative cancer treatments. These findings pave the way for further exploration of plant RiPPs, emphasizing their role in addressing the ongoing challenges in oncology and enhancing the repertoire of effective anticancer therapies.

## 1. Introduction

Cancer remains a major global health challenge, ranking as the second leading cause of death worldwide [1,2,3]. Despite significant advancements in early detection, diagnosis, and treatment, cancer remains a challenging threat to human health due to its inherent complexity [4,5]. The disease is marked by uncontrolled cell proliferation, the capacity to invade surrounding tissues, and, in many cases, the potential to metastasize to distant organs [6,7]. Furthermore, the development of drug resistance poses a significant challenge in cancer therapy [8,9]. Cancer cells can adapt and evolve, ultimately diminishing the efficacy of standard treatments over time. This resistance often results in relapse and disease progression, underscoring the urgent need for new therapeutic compounds [10,11,12,13].

Given these challenges, there is an urgent need to discover and develop novel anticancer drugs that are more effective, less toxic, and capable of overcoming drug resistance. Phytochemicals have historically served as a valuable source of therapeutic agents [14,15,16,17]. Several drugs currently employed in cancer treatment, such as paclitaxel (Taxol) [18,19,20,21,22] and vinblastine [23], originate from plant sources. This underscores the significant potential of plant-derived compounds in contributing to the next generation of anticancer therapies [24].

Ribosomally synthesized and post-translationally modified peptides (RiPPs) represent a diverse and rapidly growing class of natural products that have garnered significant attention for their effective biological activities and therapeutic potential [25,26,27]. RiPPs are unique in that they are initially synthesized as precursor peptides by the ribosome, following the standard genetic code, and subsequently undergo extensive post-translational modifications (PTMs) [28,29]. These modifications are catalyzed by specific enzymes and are crucial for converting the linear precursor peptides into biologically active structures. The structural diversity of RiPPs is driven by the wide array of potential PTMs, such as cyclization, methylation, glycosylation, hydroxylation, and the formation of unusual amino acids or cross-links [30,31]. These modifications significantly alter the chemical properties of the peptides, enhancing their stability, specificity, and potency.

Plant-derived RiPPs have emerged as a particularly promising source of new bioactive compounds. Plants have developed a diverse range of chemical defenses to combat herbivores, pathogens, and environmental stressors, many of which are mediated by RiPPs. These peptides frequently exhibit potent bioactivity while remaining minimally toxic to the plants themselves, positioning them as ideal candidates for the development of new therapeutic agents, particularly in oncology [32,33,34,35,36,37]. Plant-derived RiPPs exhibit a broad spectrum of bioactivities, functioning as potent antimicrobials, insecticides, and vasorelaxants, as well as demonstrating antiviral, immunomodulatory, and notable anticancer properties. For instance, the cyclotide kalata B1, derived from *Oldenlandia affinis*, has demonstrated antimicrobial activity against *Escherichia coli* and *Staphylococcus aureus* [38,39], as well as insecticidal effects by targeting the disruption of insect gut epithelia [40]. Similarly, the orbitide segetalin A, derived from *Vaccaria hispanica*, exhibits vasorelaxant activity, making it beneficial for the treatment of cerebrovascular spasms and hypertension [41]. Another example is the burpitides lyciumin A and lyciumin B, derived from *Lycium chinense*, which have been reported to inhibit angiotensin-converting enzyme (ACE) and renin, highlighting their potential for hypertension management [42]. Additionally, RiPPs can exhibit antiviral activity by interacting with viral proteins through their unique structure, including disulfide bonds and other post-translational modifications, or by inhibiting the entry of viruses into host cells [43,44]. RiPPs also possess immunomodulatory properties that can regulate immune responses. Stimulated by cyclopeptides, RiPPs can enhance the immune system’s ability to combat infections or cancer by modulating Caspase 3, which promotes apoptosis in infected or cancerous cells [45]. These examples highlight the diverse ecological and health-related roles of RiPPs, underscoring their potential as valuable resources for the development of bioactive compounds. Thus, RiPPs often demonstrate exceptional bioactivity, including exhibiting antimicrobial, antiviral, and anticancer properties, making them highly promising candidates for drug development [25,26,32].

The study of RiPPs derived from plants is still in its early stages, but it holds great potential. Conventional cancer treatments, such as chemotherapy and radiation, often face significant challenges including drug resistance, off-target effects, and toxic side effects [9]. These limitations underscore the need for novel, targeted therapies. Unlike traditional chemical drugs, some RiPPs exhibit high specificity and potent anticancer activity with unique mechanisms of action, making them attractive candidates for overcoming some of the limitations associated with current therapies [46,47,48]. RiPPs are naturally derived, structurally diverse peptides that undergo PTMs, enabling them to target multiple cellular pathways, such as inducing apoptosis, inhibiting angiogenesis, and modulating immune responses [49]. Their selective action and ability to be engineered for specific therapeutic purposes position them as an effective alternative to conventional anticancer drugs, offering a targeted approach with potentially fewer side effects [50].

Advances in genomics, bioinformatics, and synthetic biology are accelerating the discovery and characterization of new RiPPs, while improved techniques in structural biology are providing detailed insights into their mechanisms of action. As researchers continue to explore the rich diversity of plant RiPPs, these peptides offer new solutions to some of the most pressing challenges in cancer therapy, including the need for more effective and selective anticancer phytochemicals. In this review, we first offer a comprehensive overview of anticancer plant-derived RiPPs, emphasizing their chemistry, bioactivity, and biosynthesis. This introduction highlights that plant-derived RiPPs are a fascinating and versatile class of natural products, distinguished by their unique biosynthesis and structural complexity. The potential of RiPPs, particularly those derived from plants, represents an exciting frontier in the search for novel anticancer phytochemicals [33,35,48,51,52,53,54].

## 2. Biosynthesis of Plant-Derived RiPPs

**Figure 1:**
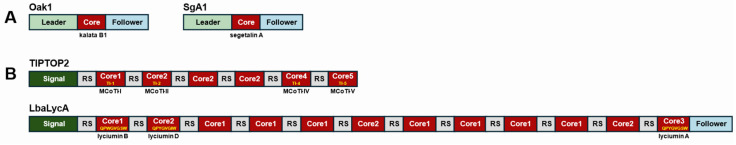
Typical biosynthetic pathway of RiPPs. The RiPP-related gene in DNA is transcribed into mRNA, and the mRNA is subsequently translated by ribosomes, resulting in the formation of a precursor peptide. (**A**) In most cases, precursor peptides are single-core form, containing a leader and core peptide, with the follower peptide being an optional component. (**B**) While less prevalent, some precursor peptides are multi-core form, consisting of two or more core peptides linked by recognition sequences. The precursor peptide undergoes post-translational modification by specific enzymes, which introduce modifications such as cyclization, dehydration, and methylation to the core peptide. Once these modifications are complete, proteases remove the leader and other peptides, converting the modified core peptide into a mature RiPP. At this stage, a variety of mature RiPPs can be produced from the multi-core precursor peptide.

Plant-derived peptides exhibit a range of physiological activities, such as antibacterial, anticancer, and antiviral properties, playing key roles in various beneficial biological processes [55,56,57]. Most known plant-derived peptides to date are produced through a biosynthetic process involving ribosomal synthesis followed by extensive post-translational modifications, classifying them as RiPPs (Figure 1) [58]. These processes allow plant-derived RiPPs to exhibit diverse structures and biological activities, such as anticancer properties, and highlight the significance of the RiPPs in therapeutic applications [59,60].

### 2.1. Ribosomal Synthesis of Precursor Peptides

Plant-derived RiPPs initiate their biosynthesis within the cellular ribosomes as precursor peptides. These precursor peptides are composed of distinct regions, which include the leader peptide, core peptide, and follower peptide, commonly referred to as single-core structures. The leader peptide is typically positioned at the N-terminus of the precursor peptide and ranges in length from approximately 20 to 110 amino acid residues. This peptide region plays a crucial role in the biosynthetic process, functioning primarily as a signal for the activation of various biosynthetic enzymes [61,62]. Furthermore, it aids in the recognition of the core peptide by these enzymes, ensuring the subsequent modifications required for maturation. Central to the precursor peptide is the core peptide, which is the key region responsible for the eventual formation of mature RiPPs through a series of enzymatic modifications. This core peptide undergoes a variety of PTMs that enhance its stability and biological activity, ultimately defining the characteristics of the final RiPPs [63].

**Figure 2:**
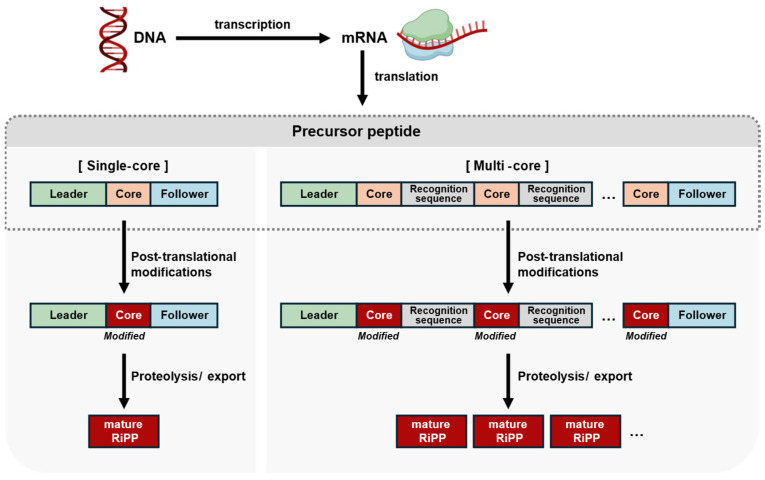
Precursor peptides of several plant-derived RiPPs. Single-core precursor peptides containing one core peptide. The core peptides of Oak1 and SgA1 are subsequently processed into kalata B1 and segetalin A, respectively. Multi-core precursor peptides containing multiple core peptides. TIPTOP2 contains six core peptides, which correspond to four types of core peptides processed into MCoTI-I, MCoTI-II, MCoTI-IV, and MCoTI-V. LbaLycA contains twelve core peptides, composed of three types that will mature into lyciumin A, lyciumin B, and lyciumin D. RS: Recognition sequence.

At the C-terminus of the precursor peptide lies the follower peptide, which, while not essential for the overall biosynthesis of the RiPPs, plays a supportive role in the modification processes of the core peptide. The follower peptide assists in targeting precise sites for enzymatic modifications, thus influencing the final structure and function of the mature peptide [64]. *O. affinis* has been traditionally used in Africa to aid women during childbirth. From this plant, a single-core precursor peptide, Oak1, is transformed into kalata B1, a member of the cyclotide class of RiPPs (Figure 2) [40]. Similarly, the Chinese herb *Saponaria vaccaria* (Caryophyllaceae), known for its effects in treating amenorrhea, regulating blood flow, and promoting lactation, produces segetalin A, an orbitide-class RiPP, from the precursor peptide SgA1 (Figure 2) [65].

Some precursor peptides of plant-derived RiPPs occur in a multi-core form, containing more than two core peptides. These multi-core precursor peptides possess several distinct recognition sequences that serve to differentiate between the multiple core peptides present within the same precursor. This feature allows them to perform a function analogous to that of follower peptides, effectively guiding the modification processes that ultimately leads to the maturation of the individual core peptides into their respective RiPPs (Figure 1) [66].

The multi-core structure of these precursor peptides offers significant advantages over traditional single-core forms, particularly in terms of biosynthetic efficiency. As they possess the capacity to generate multiple plant RiPPs from a single gene cluster, multi-core precursor peptides streamline the production process and enhance the diversity of bioactive compounds that can be obtained from a limited genetic framework. This becomes particularly beneficial in the context of plants with constrained genome sizes, where maximizing the output of bioactive peptides from existing genetic resources is crucial [67]. For instance, the precursor peptide TIPTOP2 (two inhibitor peptide topologies 2), found in the Cucurbitaceae family, contains six core peptides, from which four distinct knottin-class RiPPs—MCoTI (*Momordica cochinchinensis* trypsin inhibitor)-I, MCoTI-II, MCoTI-IV, and MCoTI-V—are produced (Figure 2) [68,69]. Similarly, in the Chinese herb *Lycium barbarum* (Solanaceae), known for its effectiveness in treating hypertension, the precursor peptide LbaLycA contains twelve linked peptides, but only three core sequences—QPYGVGSW, QPWGVGSW, and QPYGVGIW—leading to the production of the branched cyclic peptides lyciumin A, lyciumin B, and lyciumin D (Figure 2) [70].

### 2.2. Post-Translational Modifications

The core peptide of plant precursor peptides undergoes a wide range of PTMs, including but not limited to methylation, oxidation/reduction, dehydration, cross-linking between amino acid residues, and macrocyclization [71,72,73]. These modifications are critical as they enhance both the stability and biological activity of the resulting peptides, contributing to their functional roles in various physiological processes. For instance, methylation influences the charge and hydrophobicity of the peptide [74], while macrocyclization often results in a more stable structure that is less susceptible to enzymatic degradation [75].

The leader peptide plays a pivotal role in facilitating these PTMs, as it enhances the binding affinity between the core peptide and the various enzymes responsible for the modifications [61,76]. Its structural characteristics allow it to interact effectively with these enzymes, thereby ensuring that the core peptide is modified in a precise and efficient manner [77]. Moreover, the leader peptide often functions to maintain the precursor peptide in an inactive state until the modifications of the core peptide are fully completed [78]. This regulation is crucial for ensuring that the peptide remains non-functional until it has undergone the necessary alterations to become biologically active.

Follower peptides and specific recognition sequences also play a significant role in assisting PTM enzymes [48]. They provide crucial information that helps these enzymes accurately target specific sites on the core peptide for modification. For example, in the case of the plant PTM enzyme known as PCY1, it recognizes the core peptide with the assistance of a follower peptide called AYDG. This interaction is vital for catalyzing the macrocyclization of the precursor peptide, leading to the formation of orbitides, structurally characterized by their head-to-tail cyclic peptides with N-to-C amide bonds [79]. A detailed understanding of PTMs not only provides insights into the molecular mechanisms behind peptide maturation, but also opens avenues for exploring the therapeutic potentials of these modified peptides in various biomedical applications.

**Figure 3:**
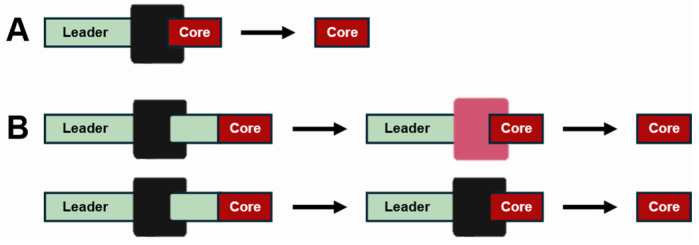
Diagram of proteolysis mechanisms in the biosynthetic pathways of plant RiPPs. (**A**) One-step removal of peptides. In this mechanism, the peptide sequence upstream or downstream of the core peptide is removed in a single step by one enzyme during maturation into the final RiPP product. (**B**) Two-step removal of peptides. In this mechanism, peptide sequences are removed through a multi-step process, either by the sequential activity of different enzymes or by repeated actions of the same enzyme.

The maturation of modified precursor peptides into RiPPs is a critical process that occurs through the proteolytic cleavage of the leader peptide and any other accompanying peptide sequences by specific enzymes known as proteases. This proteolytic removal is a finely tuned mechanism that can occur in several distinct ways. One common method is a one-step removal, where a single protease efficiently cleaves the leader peptide in one action, resulting in the immediate release of the mature RiPPs (Figure 3A) [73]. Alternatively, the process may involve a two-step removal, which can be executed either by two separate proteases acting sequentially or by a single protease performing two distinct cleavages at different sites (Figure 3B) [80]. The specificity of protease substrate recognition is a significant aspect of this maturation process, ensuring that the correct peptide sequences are cleaved at the appropriate points. For example, LahT150, a Cys protease, has been reported to remove double Gly-type leader peptides during the proteolysis of lanthipeptide, a microbial RiPP [81,82]. Currently, lantipeptides are classified as RiPPs produced by microorganisms, and the overall context of plant and microbial RiPP bio-synthesis pathways is similar [83].

### 2.3. Genetic and Biochemical Regulation of RiPP Biosynthesis

A key characteristic of plant RiPP biosynthesis is that RiPPs contain specific sequences encoding distinct functional domains. These precursor peptides undergo PTMs facilitated by various enzymes, leading to the formation of mature, functional RiPPs. While the exact mechanisms and pathways of biosynthesis differ among plant RiPP classes, much remains to be understood. Nonetheless, cyclotides and orbitides are among the most well-studied classes of plant RiPPs, with their biosynthetic processes being more thoroughly elucidated.

**Figure 4:**
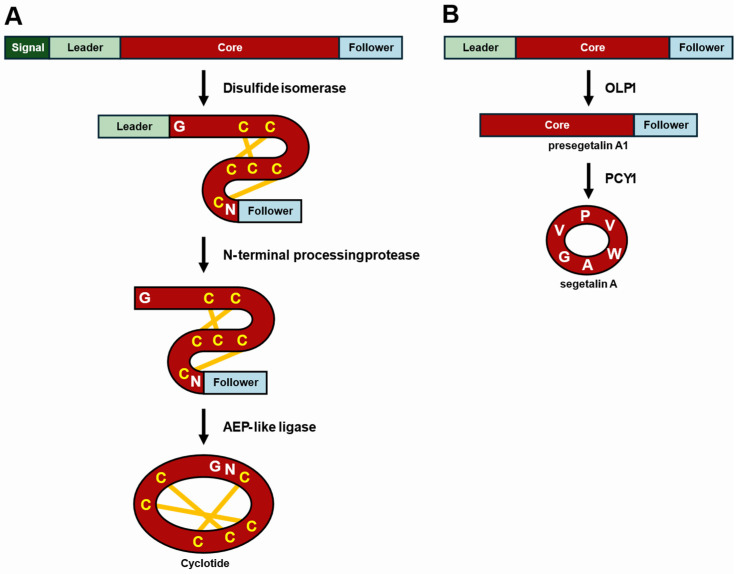
Scheme of biosynthetic pathways for a typical cyclotide (**A**) and the orbitide, segetalin A (**B**). (**A**) Cyclotide biosynthesis. The cyclotide precursor peptide is transported to the ER via an N-terminal signal sequence. Disulfide bonds are first formed by a disulfide isomerase. Subsequently, N-terminal processing proteases remove all peptides upstream of the core peptide, including the leader peptide. Finally, the head-to-tail cyclization of the core peptide is facilitated by an AEP-like ligase, which also removes the downstream peptide sequences, yielding the mature cyclotide. (**B**) Segetalin A biosynthesis. The precursor peptide for segetalin A first undergoes leader peptide removal by OLP1, resulting in presegetalin A1. Subsequently, PCY1 catalyzes macrocylization to produce the mature segetalin A.

Cyclotides, one of the most recognized classes of plant RiPPs, are head-to-tail macrocyclic peptides characterized by a “cyclic cystine knot” motif, which involves three disulfide bonds [84,85]. These bonds contribute to the remarkable structural stability of cyclotides [86]. The biosynthesis of cyclotides begins with a precursor peptide that typically contains an N-terminal endoplasmic reticulum (ER)-targeting signal sequence, an optional N-terminal leader peptide, one or more core peptide sequences, and a C-terminal follower peptide (Figure 4A). This precursor peptide is first directed to the ER via the signal sequence [87]. In the ER, it undergoes structural stabilization through folding and the formation of disulfide bonds, facilitated by disulfide isomerase [88]. This process establishes the distinct knot structure of the cyclotide, contributing to its stability and function. After stabilization, all peptides upstream of the core peptide are removed by N-terminal processing proteases, such as kalatase A, exposing the N-terminal amino acid (typically glycine) of the core peptide [89]. The final and critical step in cyclotide biosynthesis involves the head-to-tail cyclization of the peptide. This reaction is catalyzed by an asparaginyl endopeptidase (AEP)-like ligase, such as butelase 1 [90,91]. The enzyme catalyzes the formation of a bond between the N-terminal glycine and the C-terminal asparagine (or aspartate), removing the C-terminal peptide and completing the cyclization process. This step is particularly important as it contributes to the unique structural stability and bioactivity of cyclotides, which are known for their resistance to proteolytic degradation and their roles in plant defense [92,93].

Orbitides, another notable class of plant RiPPs, are smaller and simpler head-to-tail macrocyclic peptides that lack disulfide bonds [94]. The precursor peptide of an orbitide typically consists of a leader, one or more core peptides, and a follower peptide (Figure 4B). Its regulation is similar to that of cyclotides, but is less complex due to the absence of disulfide bonds [33,65,95]. The biosynthetic pathway of orbitides varies slightly depending on the type of RiPP. In the biosynthesis of segetalin A in *V. hispanica*, the peptide upstream of the core peptide in the segetalin A precursor peptide is first removed by an unidentified oligopeptidase 1 (OLP1) [79]. The resulting presegetalin A1 then undergoes macrocyclization as the C-terminal peptide is cleaved by peptide cyclase 1 (PCY1).

Understanding the specific regulatory mechanisms of plant RiPP biosynthesis can offer valuable insights into the production and discovery of promising compounds that may be utilized in the development of new therapeutics [96,97]. However, many aspects of the biosynthesis process remain to be explored, and further research appears necessary to discover new RiPPs.

## 3. Discovery of Plant-Derived RiPPs

### 3.1. Ethnobotanical Approaches and Traditional Knowledge

The discovery and identification of plant-derived RiPPs often stems from the foundational knowledge provided by ethnobotanical research, which focuses on the medicinal use of plants in traditional cultures [98]. For centuries, plants have been employed in treating various ailments, including cancer, and this historical context provides valuable insight into identifying species with potential bioactive RiPPs [99,100,101]. Ethnobotanical data are instrumental in guiding the selection of specific plants for further investigation, particularly those with documented therapeutic properties against cancer [102]. Once promising species are identified, their compounds are isolated and subjected to biological activity screening. This targeted approach, informed by traditional knowledge, enables researchers to prioritize species and extracts with a higher probability of yielding biologically active RiPPs, thereby enhancing the efficiency of discovering therapeutically relevant peptides [103,104].

### 3.2. High-Throughput Screening and Bioassay-Guided Isolation

High-throughput screening (HTS) and bioassay-guided isolation are essential methodologies in uncovering plant-derived RiPPs with anticancer potential [105]. These techniques enable efficient identification and isolation of bioactive phytochemicals from complex plant extracts, significantly advancing the discovery of new phytochemicals. HTS serves as a powerful tool, allowing researchers to rapidly evaluate the biological activity of large libraries of compounds, including plant extracts, fractions, or purified substances. The value of HTS lies in its ability to simultaneously screen thousands to millions of samples against a specific biological target, such as cancer cell lines, within a relatively short timeframe, making it indispensable in the field of compound discovery [106].

HTS usually relies on automation to efficiently handle the vast number of samples and assays involved. Robotic systems are employed for sample preparation, mixing, and dispensing into microtiter plates, which typically contain 96, 384, or 1536 wells. Each well contains a small volume of plant extract or fraction along with the designated biological target, such as cancer cells or enzymes, for screening. Detection methods in HTS, including fluorescence, luminescence, absorbance, and radioactivity, are selected based on the assay type [107]. For instance, fluorescent markers are frequently used to measure cell viability, where a reduction in fluorescence signifies cytotoxic effects on cancer cells. In cases where specific plants are already known to produce RiPPs from prior phytochemical studies, HTS can be applied to screen various plant extracts or purified fractions for their ability to inhibit cancer cell proliferation, induce apoptosis, or target cancer-specific pathways. By combining HTS with bioassay-guided isolation, researchers can adopt a comprehensive approach to identify novel RiPPs, presenting promising leads for the development of new anticancer therapies [108].

### 3.3. Genomic and Bioinformatic Approaches

Recent advancements in genomic and bioinformatic technologies have revolutionized the discovery of plant-derived RiPPs [109]. These cutting-edge approaches enable researchers to investigate the genetic information of plants, uncovering the specific genes and pathways involved in RiPP biosynthesis. Through comprehensive analysis of plant genomes and transcriptomes, biosynthetic gene clusters (BGCs) responsible for RiPP production can be identified, with predictions about the structure and function of the specific RiPPs [110,111]. Genome sequencing across various plant species, particularly those used in traditional medicine, has led to the discovery of novel RiPP BGCs that may have remained unidentified through conventional bioassay-guided methods. This genomic approach is especially valuable for finding RiPPs with anticancer potential, as each plant species harbors unique BGCs that produce structurally diverse and biologically potent peptides [112]. Advanced bioinformatics platforms, such as antiSMASH, PRISM, and RiPPER, further enhance this process by predicting and annotating RiPP BGCs, significantly streamlining the discovery of new bioactive peptides [113,114,115]. Moroidins, a newly characterized class of plant RiPPs, exhibit a unique biosynthetic mechanism involving a copper-dependent BURP domain, KjaBURP, which catalyzes their macrocyclization [35]. Notably, moroidin peptides differ from other BURP-domain-derived RiPPs, as they feature a histidine at the C-terminus instead of a tyrosine or tryptophan, resulting in distinct imidazole-indole cross-links.

Genomic approaches not only help in identifying gene clusters but also provide insights into the regulation and expression of specific RiPP BGCs [116]. Advanced techniques like RNA sequencing allow researchers to examine gene expression profiles across different plant tissues or under varying environmental conditions. By understanding the expression of RiPP BGCs, researchers can optimize experimental conditions for RiPP production in heterologous systems [117]. This knowledge is crucial for scaling up the production of RiPPs, facilitating further biological testing, and advancing their development as potential therapeutics.

## 4. Anticancer RiPPs from Plants

### 4.1. Anticancer RiPPs from the Caryophyllaceae Family

The Caryophyllaceae family, commonly referred to as the carnation or pink family, encompasses around 90 genera and over 2000 species of flowering plants. Many species within this family are widely cultivated as ornamental plants, some have also garnered scientific attention for their bioactive properties. Among these, certain species produce RiPPs, which have demonstrated significant therapeutic potential. Notably, these RiPPs have been found to exhibit anticancer properties, with the ability to suppress cancer cell growth and trigger apoptosis. This has positioned plants from the Caryophyllaceae family as valuable natural sources in the discovery for physiologically active phytochemicals, particularly in the development of novel anticancer lead compounds [118,119,120].

**Figure 5:**
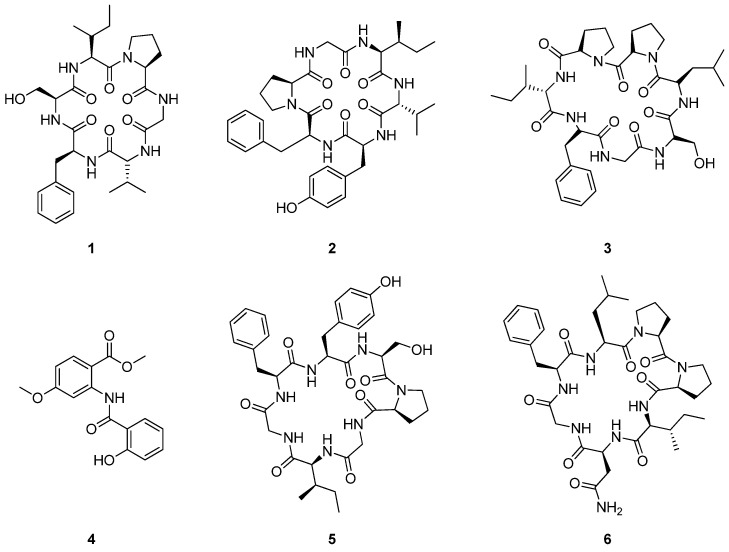
Chemical structures of compounds **1**–**6**.

*Dianthus superbus* L. is a pinkish herbaceous perennial plant growing up to 80 cm in height. It is distributed in Europe, northern Asia, France, and Japan. *D. superbus*, a plant from the Caryophyllaceae family, is commonly utilized in traditional Chinese medicine, particularly as a diuretic and anti-inflammatory agent. It is traditionally employed in the treatment of urinary infections, carbuncles, and carcinoma of the esophagus [121,122]. Phytochemical investigation on the methanolic extract of the dried and powdered whole plants of *D. superbus* led to the isolation of four phytochemicals (Figure 5). The cytotoxic activities of all identified compounds (**1**–**4**) were evaluated against five cancer cell lines: HepG2 (hepatocellular carcinoma), Hep3B (hepatocellular carcinoma), MCF-7 (breast adenocarcinoma), A549 (lung cancer cell line), and MDA-MB-231 (breast cell line), with doxorubicin serving as the positive control. The IC_50_ values (μg/mL) were used to evaluate the cytotoxic efficacy of all isolated compounds. Among the compounds tested, dianthin E (**1**) exhibited the highest cytotoxicity against the HepG2 cell line, with an IC_50_ value of 2.37 μg/mL, followed by 4-methoxydianthramide B (**4**), which showed moderate activity against 4.08 μg/mL (HepG2) and 16.02 μg/mL (Hep3B). Dianthin C (**2**) displayed weak cytotoxicity against 17.17 μg/mL (HepG2), while dianthin D (**3**) demonstrated no significant cytotoxic effects, as its IC_50_ values were greater than 20 μg/mL for all cell lines tested [123].

*Stellaria yunnanensis* Franch. is native to the Yunnan and Sichuan regions of China, flourishing in temperate environments. This perennial plant grows in shaded, moist areas, featuring long stems that extend over significant distances. Previous phytochemical investigations from the plant have uncovered several bioactive compounds in its root extracts, including cyclic peptides like yunnanin A, B, and C [124]. These peptides, particularly yunnanin C (**5**), have demonstrated potent cytotoxic activities. Yunnanin C (**5**) showed strong inhibitory effects against murine leukemia cells (P-388), with an IC_50_ value of 2.2 μg/mL (Figure 5). Additionally, it has shown antiproliferative activity against various cancer cell lines, including J774.A1 and WEHI-164, with IC_50_ values ranging from 2.1 to 7.5 µg/mL. Interestingly, synthetic analogs of these peptides, which involve slight modifications, did not exhibit the same level of cytotoxicity, potentially due to conformational changes in proline residues during synthesis [125,126].

*Dianthus chinensis* L., commonly known as the China pink or rainbow pink, is an herbaceous perennial plant and has been widely distributed to various regions of East Asia, including northern China, Korea, Mongolia, and southeastern Russia. While *D. chinensis* has not been extensively documented for medicinal use, related species in the Dianthus genus have been used in traditional medicine for their potential anti-inflammatory, antioxidant, and antimicrobial properties [127,128]. Phytochemical investigation on *D. chinensis* has led to the discovery of a class of proline-containing cyclic peptides, known as dianthiamides, and commonly referred to as orbitides. The identification and structural elucidation of dianthiamides A–E were achieved by the analysis of nuclear magnetic resonance (NMR) and mass spectrometry (MS) data [129]. Notably, dianthiamide A (**6**) exhibited cytotoxic effects against the A549 human lung cancer cell line, with an IC_50_ value of 47.9 µM, signifying a moderate ability to inhibit the proliferation of these cancer cells (Figure 5) [129].

### 4.2. Anticancer RiPPs from the Rubiaceae Family

**Figure 6:**
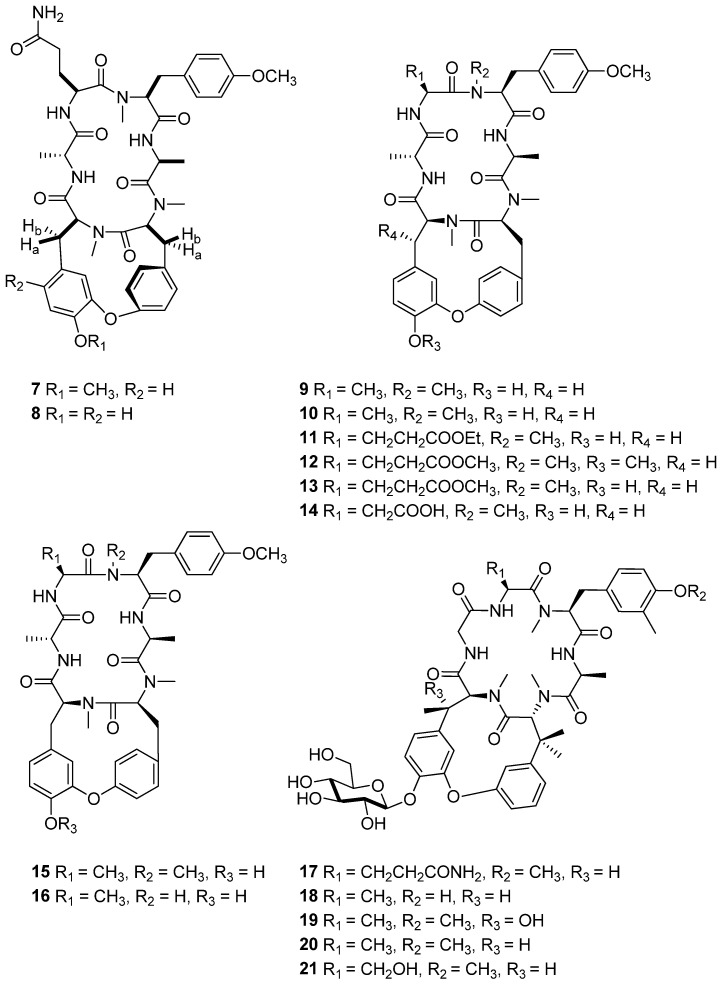
Chemical structures of cyclic peptides **7**–**21**.

*Rubia cordifolia* L. is a perennial climber native to tropical and subtropical regions of Asia, including India, Nepal, and China. This plant has long been utilized in traditional folk medicine for treating various ailments such as cough, bladder and kidney stones, joint inflammation, uterine hemorrhage, and uteritis [130]. The plant is particularly rich in anthraquinones like alizarin and purpurin, compounds that not only contribute to its dyeing properties but also its medicinal effects [131,132,133,134]. Recent studies have shown that methanol extract of the dried root of *R. cordifolia* contains the cyclic hexapeptides RA-XXIII (**7**) and RA-XXIV (**8**) (Figure 6). These compounds have demonstrated notable cytotoxic activity, particularly against leukemia cells. RA-XXIII exhibited an IC_50_ value of 0.16 µg/mL and RA-XXIV showed a value of 0.48 µg/mL against P-388 leukemia cells [135]. Notably, cyclic hexapeptide RA-V has shown potential to modulate cancer-related signaling pathways, particularly targeting pathways like Wnt, Myc, and Notch [136].

*Rubia podantha* Diels is a perennial shrub native to Europe and North Africa, thriving in various habitats such as grasslands, scrub forests, and rocky areas. The roots and rhizomes of *R. podantha* have been traditionally employed in herbal medicine to address a range of digestive disorders, attributed to the plant’s mild laxative properties that facilitate digestion and alleviate constipation [137]. Additionally, phytochemical analyses suggested that extracts from the plant possess significant anti-inflammatory effects, indicating its potential utility in the management of conditions such as arthritis and muscle pain. Compounds **9**–**14** were isolated from the roots and rhizomes of *R. podantha* (Figure 6). The cytotoxic effect of compounds **9**–**14** was assessed against cancer cell lines, including MDA-MB-231, SW620 (human colon cancer cell line), and HepG2, with IC_50_ values ranging from 0.015 to 10.27 μM. The cytotoxic effect of compounds **15** and **16** was also assessed against HeLa (cervical cancer cell line), A549, and SGC-7901 (gastric cancer cell line). RA-X (**15**) showed IC_50_ values of 3.80 ± 0.17 μM for SGC-7901, 7.14 ± 0.81 μM for A549, and 7.22 ± 0.76 μM for HeLa. Rubipodanin A (**16**) showed IC_50_ values of 0.0058 ± 0.0016 μM for SGC-7901, 0.017 ± 0.0026 μM for A549, and 0.015 ± 0.0014 μM for HeLa [138,139].

*Rubia yunnanensis* Diels. is a perennial herb native to Yunnan province in China. Traditionally, its roots have been employed in Chinese medicine to address various health issues, including tuberculosis, and specifically to aid in treating respiratory conditions. It is also utilized for reliving menoxenia and possesses anti-inflammatory properties beneficial for rheumatism. Furthermore, *R. yunnanensis* is effective in treating contusions and hematemesis, assisting with bruising and blood coughing, which are common in respiratory and gastrointestinal disorders [140,141,142,143]. Additionally, it supports blood production in anemia and is used to treat lipomas, benign fatty tumors, in traditional practices. Rubiyunnanins (**17**–**21**) isolated from the plant exhibit significant bioactivity, including cytotoxic effects against various cancer cell lines and the ability to inhibit nitric oxide (NO) production, which is associated with inflammation (Figure 6). They also suppress the NF-κB signaling pathway, a critical regulator of inflammation, immune responses, and cancer, positioning them as promising candidates for anti-cancer and anti-inflammatory therapies. Among the compounds, compound **10** showed exceptional cytotoxicity, with IC_50_ values as low as 0.01 μM against several cancer cell lines, including HepG2 (human hepatocellular carcinoma) and A549 (non-small cell lung carcinoma). This compound exhibits particularly potent activity in MDA-MB-231 (human breast carcinoma) and B16 (murine melanoma) cell lines, where it achieves IC_50_ values of 0.01 μM and 0.001 μM, respectively, highlighting its broad-spectrum antitumor efficacy. The low IC_50_ values suggest that **17** may interact effectively with critical cellular pathways, potentially leading to apoptosis in cancer cells while sparing normal cells [144].

### 4.3. Anticancer RiPPs from the Euphorbiaceae Family

**Figure 7:**
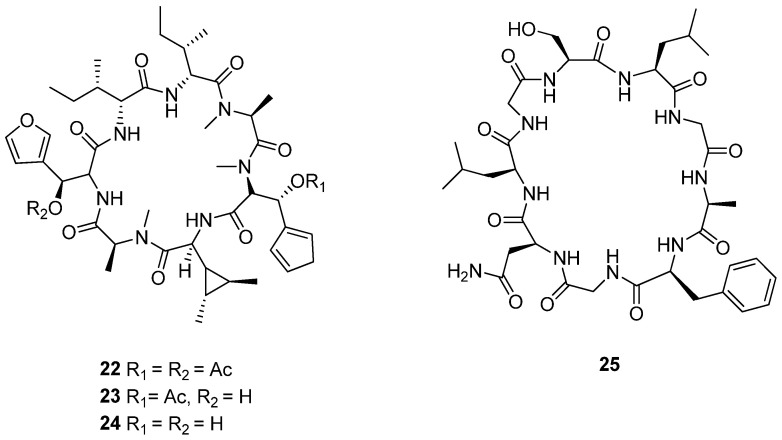
Chemical structures of cyclic peptides **22**–**25**.

*Mallotus spodocarpus* Airy Shaw. is native to Southeast Asia, particularly Thailand. Traditionally, the roots of the plant are often processed into a fine powder and applied topically to the skin to lighten pigmentation, treat dark spots, and enhance overall skin complexion. In addition to its cosmetic applications, *M. spodocarpus* has been employed in traditional remedies for various ailments, such as wound healing and treating skin conditions [145]. The plant’s extract is also believed to possess antioxidant properties, which contribute to its effectiveness in promoting skin health and potentially preventing skin damage. Recent studies have explored the phytochemical composition of *M. spodocarpus*, revealing the presence of various bioactive compounds, including flavonoids and tannins, which may enhance its medicinal properties. Mallotumides A–C (**22**–**24**) were isolated and structurally characterized from the roots of *M. spodocarpus* (Figure 7). These compounds demonstrate potent cytotoxic activity against various cancer cell lines, including KKU-M213 (intrahepatic cholangiocarcinoma), FaDu (squamous cell carcinoma), HT-29 (colon carcinoma), MDA-MB-231 (human breast adenocarcinoma), A549 (lung carcinoma), and SH-SY5Y (neuroblastoma). The cytotoxic effects of mallotumides A–C were notably strong, with IC_50_ values ranging from 0.60 to 4.80 nM across all tested cell lines, highlighting their remarkable potential to inhibit cell proliferation [146].

*Croton urucurana* Baillon, native to the tropical and subtropical regions of South America, is commonly found in Argentina, Paraguay, Bolivia, and Brazil. In traditional medicine, *C. urucurana* has been used for its antimicrobial properties, often employed to treat infections due to its effectiveness against various pathogens. The plant’s extracts have shown significant antimicrobial activity, supporting its historical use as a remedy for infections [147,148,149]. Additionally, the leaves and bark of the plant have been utilized for their anti-inflammatory effects. This characteristic makes the plant beneficial in treating conditions such as arthritis and other inflammatory diseases. Its extracts are thought to alleviate inflammation and associated discomfort. In a previous phytochemical study, [1-9-NαC]-crourorb A1 (**25**) was isolated from a 95% ethanol extract of *C. urucurana* (Figure 7). The compound was assessed for its cytotoxic activity against six human cancer cell lines: 786-0 (kidney carcinoma), HT-29 (colon carcinoma), MCF-7 (breast adenocarcinoma), NCI-ADR/RES (ovarian adenocarcinoma with multidrug resistance), Hep-G2 (liver carcinoma), and PC-03 (prostate carcinoma). The GI_50_ values, indicating the concentration required to inhibit 50% of cell growth, were as follows: Hep-G2 (41.31 ± 2.70 μg/mL), HT-29 (37.28 ± 0.57 μg/mL), MCF-7 (35.49 ± 2.59 μg/mL), PC-03 (29.80 ± 0.34 μg/mL), 786-0 (18.69 ± 0.82 μg/mL), and NCI-ADR/RES (3.98 ± 0.20 μg/mL) [150].

### 4.4. Anticancer RiPPs from the Miscellaneous Families

**Figure 8:**
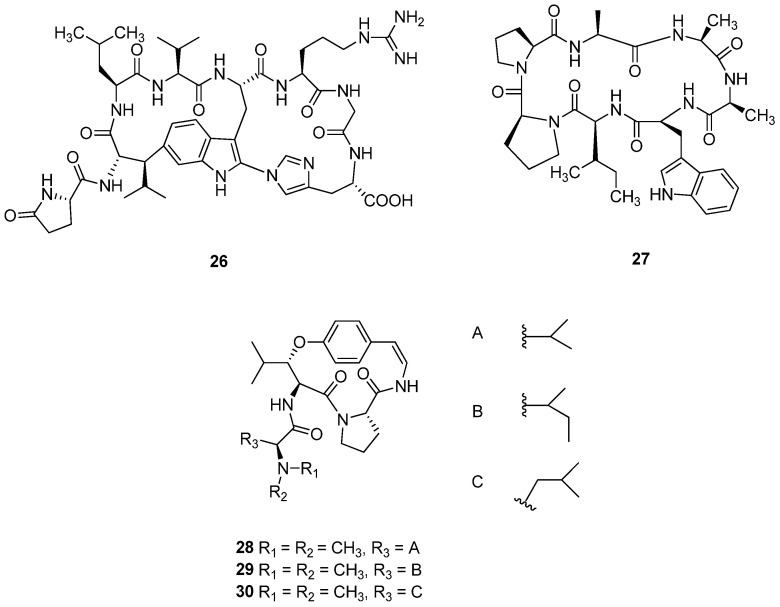
Chemical structures of cyclic peptides **26**–**30**.

*Celosia cristata* L. (Amaranthaceae) is a medicinal herb traditionally employed to treat various ailments, including fatigue, atherosclerosis, leucorrhea, and osteoporosis. This annual plant, originating from tropical regions, is characterized by its herbaceous structure, which lacks a woody stem [151,152,153,154]. From the butanol-soluble fraction of a 70% ethanol extract of *C. cristata* seeds, the cytotoxic moroidin (**26**) was purified and structurally characterized (Figure 8). The cytotoxic effects of compound **26** were tested against six cancer cell lines: A549 (lung), H1299 (lung), U87 (human brain), U251 (human brain), HCT116 (human colon), and MCF-7 (breast). The results demonstrated that **26** displayed significant cytotoxicity against A549 lung cancer cells, with an IC_50_ value of 3.2 ± 0.5 μM. Additionally, it exhibited moderate cytotoxic effects against the H1299 (IC_50_ = 8.3 ± 0.7 μM), U87 (IC_50_ = 9.6 ± 1.8 μM), U251 (IC_50_ = 5.2 ± 0.8 μM), and HCT116 (IC_50_ = 9.9 ± 1.7 μM) cell lines. However, it was less effective against MCF-7 breast cancer cells [155].

*Annona cherimola* Miller. (Annonaceae), commonly known as cherimoya, is a species of fruit-bearing tree native to the Andean valleys of Ecuador, Peru, and Colombia that is now also cultivated in various tropical and subtropical regions. In traditional medicine, *A. cherimola* has been used for various purposes. Indigenous peoples in South America have utilized the leaves and bark for their potential therapeutic effects [156,157]. Traditionally, the leaves have been employed to treat digestive issues and respiratory ailments, while the seeds are known for their insecticidal properties. Moreover, the fruit is highly nutritious, rich in vitamins, minerals, and antioxidants, which may contribute to overall health and wellness. Cherimolacylopeptide C (**27**) was isolated from dried and ground seeds of *A. cherimola* (Figure 8). The cytotoxic effect of compound **27** was evaluated against KB cells (oral cancer cell line), with IC_50_ values of 0.072 μM [158].

*Heisteria parvifolia* Sm. (Olacaceae) is native to the Amazon rainforest, primarily found in countries such as Brazil, Colombia, and Peru. The leaves and bark of the plant have often been employed in folk remedies, believed to possess anti-inflammatory and analgesic properties. Traditionally, preparations made from the plant have been used to treat ailments such as fevers, gastrointestinal issues, and skin conditions. Cycloheisterin B (**28**), cycloheisterin D (**29**), and anorldianine (**30**) were isolated from the chloroform fraction of the leaves of *H. parvifolia* (Figure 8). The anti-proliferative activities of these cyclopeptide alkaloids were evaluated against K562 cells, a human chronic myeloid leukemia cell line. Compounds **28**–**30** exhibited significant anti-proliferative effects, with cell growth inhibition rates of 46%, 44%, and 43%, respectively, at a concentration of 100 μM [159].

**Figure 9:**
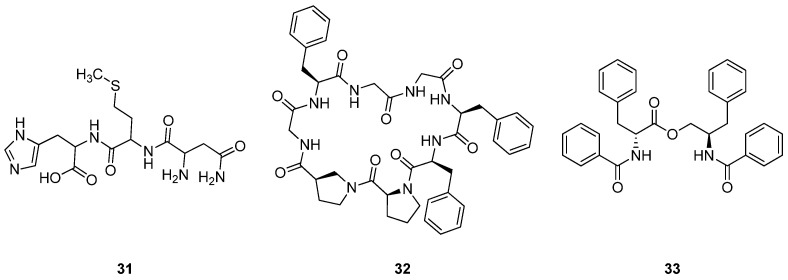
Chemical structures of peptides **31**–**33**.

*Thymus musilii* Velen (Lamiaceae) is a rare plant species primarily found in the Ha’il region of Saudi Arabia and is traditionally used in Mediterranean folk medicine. Local populations utilize the leaves and flowers of *T. musilii* as garnishes, in teas, and for treating various microbial diseases, particularly valuing its antimicrobial and anti-inflammatory properties [160,161,162]. A tripeptide (**31**) isolated from the methanolic extract of *T. musilii* exhibited significant cytotoxic effects, with IC_50_ values of 107.69 µg/mL against MCF-7, 153.54 µg/mL against HCT-116, and 194.70 µg/mL against A549 cell lines (Figure 9). Additionally, its docking score of −8.983 kcal/mol indicates a strong binding affinity to the MLK4 kinase domain (4UYA), suggesting its potential to disrupt critical cancer signaling pathways such as JNK and ERK [161].

*Zanthoxylum riedelianum* L. (Rutaceae) is widely distributed in South America, particularly found in Brazil and surrounding regions. The barks, leaves, and fruits of *Z. riedelianum* have been utilized for their potential medicinal properties, including anti-inflammatory, analgesic, antifungal, and antimicrobial effects [148,163]. In some cultures, extracts from the plant have been used to treat gastrointestinal disorders, respiratory issues, and skin ailments. Additionally, its antiseptic properties make it valuable for wound healing and in the preparation of traditional remedies against infections. *Z. riedelianum* yields a significant cyclic peptide known as [1-8-NαC]-zanriorb A1 (**32**), which exhibits notable cytotoxic activity against leukemia T cells (Jurkat) with an IC_50_ value of 218 nM (Figure 9) [164].

*Colubrina asiatica* L. (Rhamnaceae) is a climbing shrub native to Southeast Asia, northern Australia, and the Pacific islands, reaching heights of up to 4 m and commonly found in wetlands and coastal areas. This plant has traditional medicinal applications wherein its leaves and bark are used to treat skin diseases, while the roots are utilized to alleviate fever and thirst [165,166,167]. A derivative of *N*-benzoyl-D-phenylalanine (**33**) has been found to possess weak cytotoxicity against various cancer cell lines, including HeLa cervical cancer (KB), small cell lung carcinoma (NCI-H187), and human breast cancer (MCF-7). Specifically, compound **33** exhibited notable cytotoxicity against the NCI-H187 cell line, with an IC_50_ value of 19.51 μg/mL, marking it as the only peptide in this study to demonstrate such activity (Figure 9) [168].

## 5. Apoptotic Mechanisms Induced by Plant-Derived RiPPs in Cancer Cells

Caspases, a family of cysteine proteases, are central to the initiation and execution of apoptosis. Distinct caspases mediate either the intrinsic mitochondrial pathway or the extrinsic death receptor pathway, which converge to break apart cellular components and execute programmed cell death [169,170]. Identifying the specific caspases activated by plant-derived RiPPs is crucial for the identification of their apoptotic mechanisms. By selectively engaging caspase-3, caspase-8, caspase-3/7, or caspase-9, RiPPs effectively disrupt cancer cell survival pathways, providing a mechanistic foundation for their therapeutic potential [171,172]. Furthermore, understanding the link between the chemical structure of RiPPs and their ability to activate caspases is crucial for designing and optimizing RiPP-based anticancer agents (Table 1).

Vigno 5, a cyclotide isolated from *Viola ignobilis*, induced apoptosis in HeLa by activating caspase-3 and caspase-9 [173]. The apoptosis was mediated through the mitochondrial pathway, characterized by the release of cytochrome C, upregulation of Bax, downregulation of Bcl-2, and cleavage of PARP1, ultimately leading to DNA fragmentation. Similarly, orbitides identified from *Linum usitatissimum* L. have been shown to activate caspase-3 and caspase-9 in SGC-7901 via mitochondrial depolarization and Bax/Bcl-2 ratio modulation, resulting in PARP cleavage and apoptosis [174].

[1–9-NαC]-crourorb A1, a orbitide identified from *C. urucurana* latex, activated caspase-3/7 in Huh-7 (human hepatocarcinoma cell line) [175], inducing apoptosis through G2/M cell cycle arrest and a JNK-mediated pathway. This process was accompanied by increased expression of pro-apoptotic proteins, including Bak, Bax, and Puma, highlighting its potential for targeting liver cancer.

Another orbitide, flaxseed-derived linusorbs, also exhibited significant caspase activation in various cancer cells. [1–9-NαC]-linusorb B2 activated caspase-3 and caspase-8 in HepG2 [176] and SGC-7901 [177]. In HepG2, the DR4 death receptor pathway was involved, while in SGC-7901, the Fas/FasL signaling cascade triggered apoptosis, resulting in mitochondrial dysfunction and PARP cleavage. [1–9-NαC]-linusorb B3 demonstrated a broad apoptotic profile across multiple cancer cell lines. In HepG2, it activated caspase-3 and caspase-8 via DR4-mediated apoptosis, involving Bax upregulation, cytochrome C release, and Bcl-2 downregulation [176]. In SGC-7901, it activated caspase-3, caspase-8, and caspase-9, involving dual intrinsic and extrinsic pathways [177]. Furthermore, in C6 (glioblastoma cell line), it induced caspase-3 and caspase-9 activation via mitochondrial-mediated apoptosis, characterized by mitochondrial membrane depolarization and p53 suppression [178].

Moroidin, isolated from the seeds of *C. cristata*, activated caspase-3 and caspase-9 in A549 through the intrinsic mitochondrial pathway [155]. This involved cytochrome C release, Bax upregulation, Bcl-2 downregulation, and PARP cleavage, resulting in apoptotic cell death. Moroidin is categorized as a burpitide, a subclass of RiPPs derived from or associated with the BURP domain, which plays a key role in plant development and responses to environmental stress. [179].

RA-V, a cyclopeptide isolated from *R. cordifolia*, selectively activated caspase-3 in Kras-dependent non-small-cell lung carcinoma (NSCLC) cell lines (H441 and H358) [180]. This apoptotic activity was mediated by TAK1 inhibition, leading to reduced expression of anti-apoptotic proteins such as Bcl-2 and Bcl-XL. RA-V from *R. yunnanensis* also activated caspase-3 and caspase-9 in breast cancer cell lines (MCF-7 and MDA-MB-231) [181], with apoptosis mediated by mitochondrial dysfunction, cytochrome C release, and PARP cleavage. Additionally, RA-XII, another cyclopeptide from *R. yunnanensis*, induced caspase-3, caspase-8, and caspase-9 activation in HepG2 through Bax upregulation, Bcl-2 downregulation, and cytochrome C release, ultimately leading to mitochondrial-mediated apoptosis [182].

**Table 1:** Types of caspases activated by plant-derived RiPPs for cancer cells.

Compounds	Type	Source (Plant)	Target	Cell Line	Ref.
Vigno 5	Cyclotide	*Viola ignobilis*	Caspase-3, -9	HeLa	[173]
Flaxseed orbitides (extract)	Orbitide	*Linum usitatissimum* L.	Caspase-3, -9	SGC-7901	[174]
[1–9-NαC]-crourorb A1	Orbitide	*Croton urucurana*	Caspase-3/7	Huh-7	[175]
[1–9-NαC]- Linusorb B2	Orbitide	*L. usitatissimum* L.	Caspase-3, -8	HepG2	[176]
			Caspase-3, -8	SGC-7901	[177]
[1–9-NαC]- Linusorb B3	Orbitide	*L. usitatissimum* L.	Caspase-3, -8	HepG2	[176]
			Caspase-3, -8, -9	SGC-7901	[177]
			Caspase-3, -9	C6	[178]
Moroidin	Burpitide	*Celosia cristata*	Caspase-3, -9	A549	[155]
RA-V	Undefined	*Rubia cordifolia*	Caspase-3	H441	[180]
			Caspase-3	H358	[180]
		*R. yunnanensis*	Caspase-3, -9	MCF-7	[181]
			Caspase-3, -9	MDA-MB-231	[181]
RA-XII	Undefined	*R. yunnanensis*	Caspase-3, -8, -9	HepG2	[182]

## 6. Challenges in the Development of Plant-Derived RiPPs as Anticancer Agents

### 6.1. Complexity of RiPP Biosynthesis

A significant challenge in developing plant-derived RiPPs lies in the complexity of their biosynthetic processes. RiPPs are produced from precursor peptides through a series of enzymatic modifications, which can differ greatly across plant species. This variability is due to the diversity of BGCs in different plants, resulting in structural variations in RiPPs. These variations are driven by evolutionary forces that adapt biosynthetic pathways for advantages such as increased pathogen resistance or better ecological interactions. As a result, discovering and characterizing these BGCs is a critical yet difficult task in the search for novel anticancer RiPPs [54].

RiPPs undergo extensive post-translational modifications (PTMs) following their initial ribosomal synthesis, adding complexity to the identification and characterization of plant-derived RiPPs [48]. Although different plants may produce similar precursor peptides, the modifications they undergo can vary significantly, leading to distinct biological activities. Understanding these modifications is essential for harnessing the therapeutic potential of plant-derived RiPPs. Many plant-derived RiPPs possess cyclic structures that enhance their stability and resistance to enzymatic breakdown, contributing to stronger interactions with biological targets. These PTMs, such as methylation, hydroxylation, and phosphorylation, affect the peptide’s charge, hydrophobicity, and structure, ultimately influencing its affinity and specificity for binding to cellular receptors or enzymes, and improving its therapeutic efficacy [183,184].

The complexity of RiPP biosynthesis presents both challenges and opportunities for the development of plant-derived RiPPs as anticancer agents [185]. While the diverse PTMs and genetic variability complicate the identification and characterization of their chemical structures, they also provide a rich source of novel compounds with unique structures and mechanisms of action. By leveraging advanced genomic, proteomic, and metabolomic approaches, researchers can unlock the potential of RiPPs, optimizing their biosynthesis for therapeutic applications and ultimately paving the way for the development of new cancer treatments [186].

### 6.2. Low Yield and Purification Challenges

Identification of plant-derived RiPPs as anticancer agents is significantly hindered by the low yield and purification challenges. Many plant-derived RiPPs occur in minute quantities within their natural plant hosts, which poses a considerable challenge for their isolation and identification. RiPPs are classified as secondary metabolites, which are not essential for the primary growth and reproduction of plants. Plants often produce them in response to specific environmental stimuli, such as defense against pathogens, herbivores, or stress [187]. Consequently, RiPP production tends to be inconsistent and varies depending on external factors. This makes it difficult to predict or control the amounts of RiPPs that can be extracted from plants under natural conditions. The BGCs responsible for RiPP biosynthesis in plants are often expressed at low levels or are activated only under specific conditions. As a result, even though a plant may possess the genetic capacity to produce a RiPP, the actual quantities synthesized may be insufficient for practical extraction and subsequent chromatographic isolation. This variability further complicates the production of RiPPs in quantities suitable for pharmaceutical research and development [188].

Plant tissues contain a wide array of biomolecules, including proteins, polysaccharides, lipids, and secondary metabolites, which complicate the isolation of RiPPs [47,186]. These compounds interfere with extraction and purification processes, making it challenging to isolate RiPPs. Moreover, RiPPs are often chemically unstable and susceptible to degradation when exposed to harsh extraction conditions, such as organic solvents, extreme pH, or elevated temperatures [189]. This instability results in the breakdown or modification of RiPPs, leading to diminished yields and loss of biological activity. Purification typically involves multiple chromatography steps, which are labor-intensive and costly [190]. The low yield of RiPPs from plants, combined with the complex purification protocols required, leads to significant losses and further reduces the overall efficiency of RiPP production.

While *D. chinensis* has demonstrated promising anticancer activity due to its RiPP-derived peptides, the yield of these compounds remains a challenge for large-scale production [191]. Additionally, toxicity concerns associated with certain peptides may limit their therapeutic application in clinical settings [192,193]. Similarly, *R. cordifolia* showed potential in traditional medicine, but the complexities involved in extracting and optimizing RiPPs for therapeutic use, alongside issues of limited yields and potential toxicity, need to be carefully addressed for future drug development [194].

Several strategies are being explored to overcome the low yield and extraction challenges associated with plant-derived RiPPs. Among the approaches, heterologous expression systems offer an alternative method for producing RiPPs in higher yields [195]. By transferring the BGCs responsible for RiPP production into microbial hosts, such as bacteria or yeast, researchers bypass the need for direct extraction from plants. These microbial systems can be engineered to overproduce RiPPs in controlled conditions, thereby increasing the yield and simplifying the purification process. Synthetic biology approaches that optimize RiPP BGCs for expression in heterologous hosts are being actively explored to address this challenge [196].

## 7. Conclusions

The exploration of plant RiPPs represents a significant advancement in the discovery of novel anticancer agents. This innovative field of research merges centuries of traditional ethnobotanical knowledge, derived from the medicinal use of plants, with cutting-edge genomic and bioinformatic technologies. By harnessing insights from ethnobotanical studies, researchers have identified plant species with documented therapeutic properties, thereby guiding the selection of candidates for further phytochemical investigation. This integrated approach markedly enhances the efficiency of discovering biologically active RiPPs that hold great promise for cancer treatment.

In this review, we focus on the discovery of plant RiPPs as valuable candidates for therapeutic applications in cancer treatment. While recent reviews have addressed various aspects of plant RiPPs, our emphasis lies specifically on their chemical structures and cytotoxic activities. The application of high-throughput screening (HTS) and bioassay-guided isolation methodologies has revolutionized the identification of bioactive RiPPs from complex plant extracts. These techniques facilitate the rapid evaluation of a wide array of phytochemicals, including fractions and purified RiPPs, against specific biological targets such as cancer cell lines.

Despite the promising advancements in the field of plant RiPPs as anticancer agents, several challenges impede their effective utilization. One significant hurdle lies in the inherent complexity of RiPP biosynthesis, as these peptides undergo a series of enzymatic modifications that exhibit considerable variability among different plant species. This variability in BGCs often leads to distinct structural variations among RiPPs, each associated with unique biological activities. A comprehensive understanding of these variations is essential for fully harnessing the therapeutic potential of plant RiPPs; however, this complexity complicates the identification and characterization processes. Additionally, the low yields of RiPPs and the challenges associated with their purification further exacerbate the difficulties faced in RiPP discovery. As secondary metabolites, RiPPs are typically found in minute quantities within their natural plant hosts, necessitating the development of sophisticated extraction and purification techniques to isolate these valuable compounds effectively.

To overcome these significant challenges, researchers are actively investigating innovative strategies, including heterologous expression systems and synthetic biology approaches. By transferring the BGCs responsible for RiPP production into microbial hosts such as bacteria or yeast, scientists have been able to achieve higher yields and simplify purification processes, thereby enabling more efficient production of these peptides.

Advances in synthetic biology and heterologous expression systems have significantly enhanced the potential for RiPP-based drug development. Synthetic biology facilitates the design and optimization of biosynthetic pathways, enabling the creation of novel RiPPs with tailored properties. This addresses critical challenges such as low yields and structural complexity. By manipulating BGCs, researchers can design peptides with enhanced bioactivity, stability, and specificity, thereby broadening their therapeutic applications. Moreover, heterologous expression systems, particularly when applied to plant or microbial BGCs, allow for the scalable production of RiPPs that are difficult to obtain from natural sources. These systems enable the expression of complex RiPPs in high yields, overcoming limitations associated with low natural production or host toxicity. This capability opens new avenues for pharmaceutical development, accelerating the testing and commercialization of novel RiPP-based therapeutics. As synthetic biology and heterologous expression technologies continue to evolve, the rapid and cost-effective development of RiPP-based drugs is becoming increasingly feasible. These advancements are driving transformative changes in drug discovery and biotechnology. Additionally, complementary technologies such as HTS, bioassay-guided isolation, and genomic studies play pivotal roles in overcoming existing barriers, enabling the discovery and production of more efficient RiPP-based treatments.

In conclusion, the future of plant RiPPs as anticancer agents is promising yet fraught with challenges. The ongoing collaboration between ethnobotanists, molecular biologists, and chemists is essential to navigate the complexities associated with RiPP biosynthesis and to unlock their full therapeutic potential. By combining traditional knowledge with modern scientific advancements, we may pave the way for innovative cancer treatments that not only harness the rich biodiversity of various natural sources but also contribute to the development of safer and more effective therapeutic options. Ultimately, this synergy can unlock the full potential of plant-derived RiPPs, paving the way for advancements in cancer treatment and contributing to the field of natural product research.

## Data Availability

Not applicable.
